# 

**Published:** 1918-12

**Authors:** 


### CONTENTS FOR THE DECEMBER ISSUE

EDITORIALS.

A Christmas Wish.................................... 185

A Joyful Christmas.................................... 186

A Deserved Tribute.................................... 187

The Cover Page.................................... 187

Get Rid of German Control.................................... 187

An Interesting Series of Articles.................................... 188

ORIGINAL ARTICLES.

The General Practitioner and the Health Problem. By George A. Hastings, New York.................................... 191

Tabloid Thoughts on Sleeplessness. Dr. Edwin F. Bowers, New York.................................... 197

State Tuberculosis Sanitorium Has Bureau of Correspondence and Information.................................... 197

A Letter From the Front.................................... 202

Military Anti-Tuberculosis Program Perfected.......... 208

Crippled Soldiers and Sailors to Be Taught Useful Occupations .................................... 211

Subscription Acknowledgments.................................... 213

How Wound Shock Is Cared for in France.............. 213

Army News .................................... 214

Old Jokes .................................... 216

Publisher's Department .................................... 218

